# Genomic analysis reveals epigenetic ‘addiction’ underpinning follicular lymphoma and its transformation – a rationale for targeted epigenetic therapies

**DOI:** 10.1186/1868-7083-5-S1-S10

**Published:** 2013-08-19

**Authors:** Jessica Okosun, Csaba Bödör, Jun Wang, Shamzah Araf, Claude Chelala, Jude Fitzgibbon

**Affiliations:** 1 Centre for Haemato-Oncology, Barts Cancer Institute, Queen Mary University of London, London, EC1M 6BQ, UK; 2 1st Department of Pathology and Experimental Cancer Research, Semmelweis University, Budapest, Hungary; 3 Centre for Molecular Oncology, Barts Cancer Institute, Queen Mary University of London, London, EC1M 6BQ, UK

## 

Follicular lymphoma (FL) continues to pose a clinical challenge with a progressive disease course typified by multiple relapses, eventual resistance to standard therapies and transformation (tFL) in a subset of patients to the more aggressive diffuse large B cell lymphoma (DLBCL).

WGS was performed on 6 paired FL-tFL patients and corresponding germ-lines to understand the clonal dynamics of progression and recurring genetic events in FL. We identified that the dominant pattern of FL evolution to transformation was consistent with the existence of a common origin, an ancestral common progenitor cell (CPC) population. Strikingly, the CPCs were enriched for mutations in chromatin regulatory genes with every case harbouring mutations in *MLL2*, a histone methyltransferase, concurrent with mutations in other methyltransferases (*EZH2*, *MLL3*, *PRMT2*), acetyltransferases (*CREBBP*, *MEF2B*), bromodomain proteins (*BRD2*, *CECR2*), core and linker histones. Targeted deep sequencing of *EZH2* showed mutations in 27% of cases in an extension cohort of 366 cases, much higher than previously reported. All mutations targeted the catalytic SET domain leading to a gain-of-function thus offering the possibility of using EZH2 inhibitors.

The plethora of genetic mutations in epigenetic regulators in FL shown in this study therefore offers a compelling model disease to strategically test epigenetically-targeted therapies.

